# Bioenergy production from chicken feather waste by anaerobic digestion and bioelectrochemical systems

**DOI:** 10.1186/s12934-024-02374-5

**Published:** 2024-04-04

**Authors:** Dina Hassan El Salamony, Mohamed Salah Eldin Hassouna, Taha Ibrahim Zaghloul, Zhen He, Hanan Moustafa Abdallah

**Affiliations:** 1 Department of Biotechnology, Institute of Graduate Studies and Research, Alexandria University, Alexandria, Egypt; 2 Department of Environmental Studies, Institute of Graduate Studies and Research, Alexandria University, Alexandria, Egypt; 3 Department of Biotechnology, Institute of Graduate Studies and Research, Alexandria University, Alexandria, Egypt; 4 Department of Energy, Environmental and Chemical Engineering, Washington University in St. Louis, St. Louis, MO 63130 USA

**Keywords:** Microbial fuel cell, Microbial electrolysis cell, Poultry waste, Biomethane production, Bioenergy generation, Green hydrogen

## Abstract

**Background:**

Poultry feather waste has a potential for bioenergy production because of its high protein content. This research explored the use of chicken feather hydrolysate for methane and hydrogen production via anaerobic digestion and bioelectrochemical systems, respectively. Solid state fermentation of chicken waste was conducted using a recombinant strain of *Bacillus subtilis* DB100 (p5.2).

**Results:**

In the anaerobic digestion, feather hydrolysate produced maximally 0.67 Nm^3^ CH_4_/kg feathers and 0.85 mmol H_2_/day.L concomitant to COD removal of 86% and 93%, respectively. The bioelectrochemical systems used were microbial fuel and electrolysis cells. In the first using a microbial fuel cell, feather hydrolysate produced electricity with a maximum cell potential of 375 mV and a current of 0.52 mA. In the microbial electrolysis cell, the hydrolysate enhanced the hydrogen production rate to 7.5 mmol/day.L, with a current density of 11.5 A/m^2^ and a power density of 9.26 W/m^2^.

**Conclusions:**

The data indicated that the sustainable utilization of keratin hydrolysate to produce electricity and biohydrogen via bioelectrical chemical systems is feasible. Keratin hydrolysate can produce electricity and biofuels through an integrated aerobic-anaerobic fermentation system.

**Graphical Abstract:**

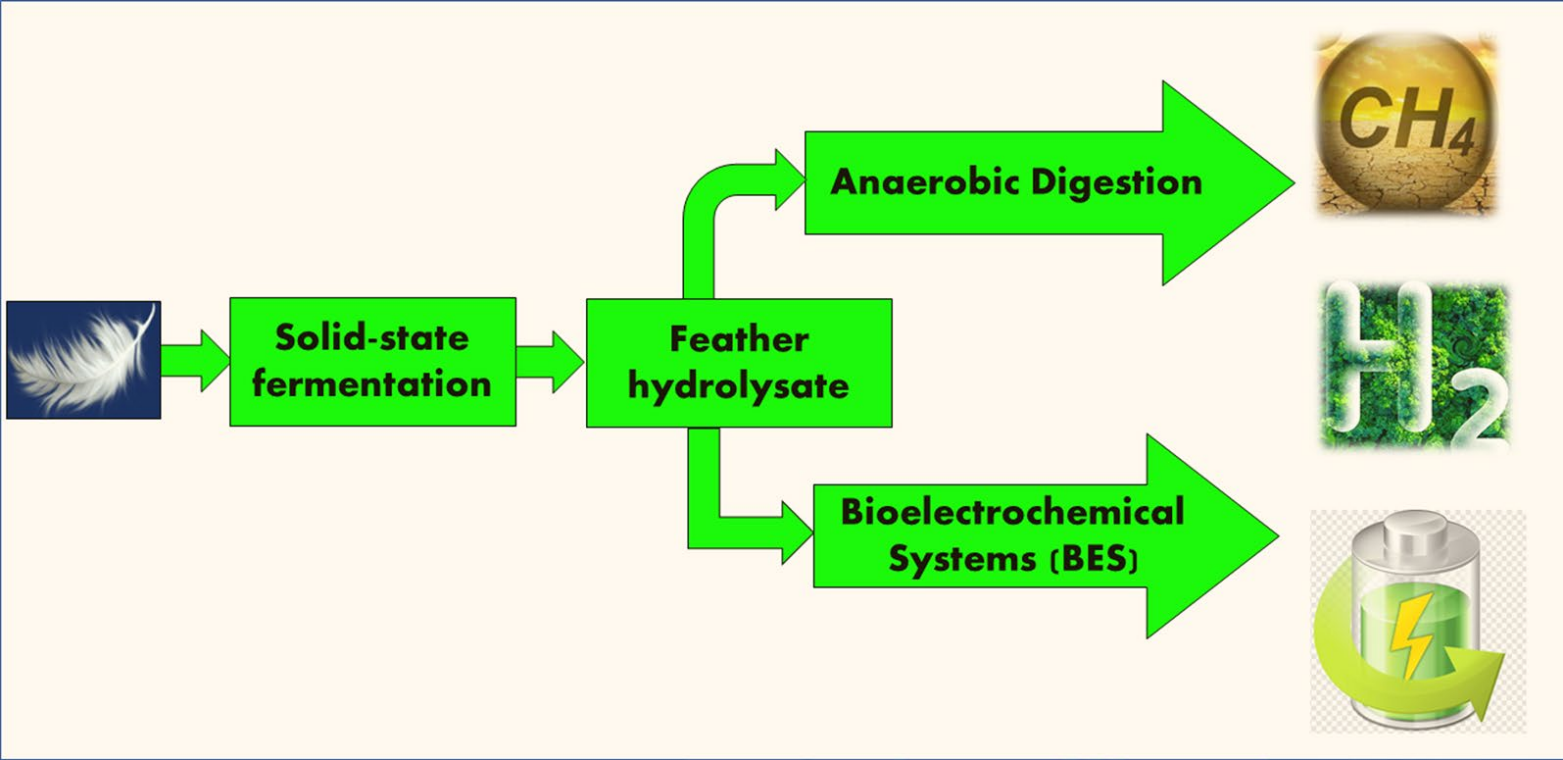

**Supplementary Information:**

The online version contains supplementary material available at 10.1186/s12934-024-02374-5.

## Background

Biorefinery is a green technology that converts organic waste into valuable products such as biofertilizers, bioethanol, biohydrogen, bioelectricity, biogas, and organic acids [1]. Scientists are exploring alternatives to reduce carbon dioxide emissions from conventional energy production [2]. Using microbial fermentation to generate bioenergy is economically and environmentally advantageous [3].

Biobased industrial byproducts, including food industry side streams, are promising alternatives with great valorization potential. The global poultry production process incinerates approximately 40 million metric tons of chicken feathers each year, releasing harmful fumes such as carbon and sulfur dioxides [4]. Thus, repurposing feather waste reduces environmental pollution and carbon emissions.

Chicken feathers with high protein and low lipid contents have potential as raw materials for valuable soluble proteins, industrial enzymes, and bioenergy production [5, 6]. Converting industrial waste into renewable energy materials at a low cost and high scalability could enhance the circular economy [7].

Recycling feather waste into animal feed is restricted due to concerns about disease transmission. Anaerobic digestion offers a viable waste valorization option. Methane, the primary component of biogas, can power vehicles, serve as an alternative transportation fuel, or be utilized in energy products [8]. Combining waste management, energy generation, and nutrient recycling through composting is another way to benefit from this waste [9]. Keratin-rich biomass mixed with other wastes, like cellulosic feedstocks and agricultural residues, high in carbon but low in nitrogen, can help maintain the balance of macronutrients to grow microorganisms [9, 10].

Electroactive bacteria in bioelectrochemical systems (BESs) break down organic waste into protons and electrons for use in anodic oxidation and reduction reactions [10]. Researchers have tested various types of waste in different types of BESs. The feasibility of using waste depends on the nature of the components present and their biodegradability [11]. Microbial fuel cells (MFCs) and microbial electrolysis cells (MECs) are two types of bioelectrochemical systems that are used to make bioelectricity and biohydrogen, as well as clean up the environment from waste [12, 13].

Microbial fuel cells use microbes to oxidize organic waste from various sources, producing electrical power [14, 15]. The core of microbial fuel cells consists of an anion exchange membrane (AEM) between the anaerobic anode chamber and the aerobic cathode chamber. Recent research indicates the utilization of complex chemicals such as oilseed cakes, which are byproducts produced during cooking oil extraction from edible oilseeds and fuel from non-edible oilseeds. These microorganisms are commonly used as nutritional additives in animal feed for chicken, swine, and dairy due to their vitamins, proteins, and ability to produce bioelectricity in MFCs. An electrogenic bacterium species was discovered through microbial fuel cells (MFC) with oilseed cake as a growth substrate [16].

A microbial electrolysis cell consists of an anode, anion exchange membrane, a catalyzed hydrogen progression cathode, and an electrolyte containing microorganisms [17]. For MECs to work, the cathode chamber must be utterly anaerobic for the chemical evolution of hydrogen to occur usually [18]. The core of an MEC consists of a microbial anode and an "almost conventional" hydrogen evolution cathode [19]. The electrohydrogenesis process helps oxidize organic compounds anaerobically in the anode chamber [20].

Biological methods such as photofermentation, dark fermentation, and green algae can produce hydrogen as a sustainable energy source [21]. Chemical, thermochemical, and electrochemical processes produce hydrogen. Depending on its source, hydrogen falls into the categories of gray, blue, or green. While carbon capture and storage systems produce blue hydrogen, hydrocarbon reformation processes, such as steam methane reforming, produce gray hydrogen. Green hydrogen is the most environmentally friendly form [13, 22, 23].

The feedstock type determines the potential for biohydrogen production in MECs, which includes several waste substrates. Research on efficient infrastructure designs, electrode materials, separators, catalysts, and genetically modified microorganisms has made BES a promising technology [24].

Scientists use a constructed fermentation approach to produce bioenergy from keratin-based wastes [25]. New methods have been developed to convert chicken feathers into sustainable, zero-waste fuel cells, for instance, by extracting protein keratin and processing it into amyloid fibrils, which are crucial components of fuel cells. Recently, researchers tested feather-based membranes by assembling them in a commercial fuel cell setup [7].

As a result, this study looked at a two-stage fermentation system that uses feathers. A previous study [6] detailed the initial steps in transforming keratinaceous biowaste into valuable products. Aerobic solid-state fermentation with recombinant *Bacillus subtilis* DB100 (p5.2) produced industrial enzymes and soluble proteins. The recombinant strain in this study contained the p5.2 plasmid (4.7 kbp), a variant of the pUB110 plasmid. The p5.2 plasmid contains the entire 750-bp alkaline protease gene, called *apr*E, which exhibits both proteolytic and keratinolytic activities. The recombinant plasmid's stability enhances the efficiency and productivity of the fermentation process in a shorter time than the native strain [26]. This study aimed to utilize feather hydrolysate for electricity generation and biofuel production via anaerobic digestion and bioelectrochemical systems as an environmentally friendly and sustainable approach for transforming waste into bioenergy.

## Materials and methods

### Elemental analysis of the feather hydrolysate

Feather hydrolysate (FH) used in this study was obtained from the first aerobic fermentation step of chicken feather waste via the recombinant strain *Bacillus subtilis* DB100 (p5.2) [27]. Energy dispersive X-ray analysis (EDX) (Joel JSM 6360 LA-, Tokyo, Japan) was used to analyze the elemental composition of the two feather hydrolysate samples. They were divided into two groups: The first was obtained from solid-state fermentation of chicken feathers alone (SSF), and the other was supplemented with wheat bran. The potency of the digestion of chicken feathers by *B. subtilis* DB100 (p5.2) was evaluated as a promising start for the next steps of anaerobic fermentation.

### Methane production via anaerobic digestion

Three different groups were used to carry out anaerobic digestion, depending on the substrate used. Acetate served as the control in the control treatment. The second treatment utilized hydrolysate derived from biologically treated chicken feathers (FH). Treatment three utilized untreated chicken feather particles (FPs). The tests were done in a mesophilic environment ( 37 ± 1 °C) in 500 mL serum glass bottles with an active volume of 300 mL [28]. Alexandria East Sewage Treatment Plant initially provided the digested sludge used in this study. An Anaerobic medium was added, as described earlier [29], with some modifications. The media was modified to contain 15 g/L peptone, 8 g/L glucose, 8 g/L yeast extract, and 0.5 g/L sodium bicarbonate. One tablet of potassium TRI B (Carlson® Tri-B, Vitamin B Complex) was also added to the stock solution of trace elements. The pH of each reactor was adjusted to be in the range of 6.8–7.2 using a hydrochloric acid solution (1 M) or sodium hydroxide (1 N). The bottles were flushed with nitrogen gas (99.998%) for 5–10 minutes and then sealed with rubber septa and aluminum caps [30]. After the reactors were incubated for 30 days, they were shaken manually once daily. Gas samples were collected from the headspace of each reactor twice a week at the beginning and once a week toward the end of the digestion period. Samples were analyzed for methane gas analysis by a gas chromatograph (GC-2014) (SHIMADSU, Kyoto, Japan) equipped with an FID detector and capillary column (Elite-5, 30 m × 0.25 mm × 0.25 μm) (flow rate 20 mL/min with column temperature of 120 °C, injection volume of 1 ml gas sample), the carrier gas was helium. The quantitative analysis and measurement of the resulting gases were carried out as described previously [30].

### Hydrogen production via anaerobic digestion

The reaction mixtures used to produce biogas were also utilized for hydrogen production. A methanogenic bacterial inhibitor (286 µM 2-bromoethanesulfonate) was added to stop biogas production [31]. Three treatments were tested: the first was fed with acetate (as the control), the second was with feather hydrolysate, and the third treatment consisted of untreated feather particles (FPs). In addition to the inhibitor used, the pH was adjusted to 8.5–9 to inhibit methanogenesis. A MQ-8 sensor was used to detect hydrogen gas that had been produced. MQ-8 is a semiconductor-type gas sensor (FUTURE Electronics Group Corporation, EGYPT) with a high sensitivity to hydrogen and a circuit voltage of 5 V [32]. Experiments were performed in triplicate, and statistical analyses were carried out using GraphPad Prism 6.00.

### Microbial fuel cell construction and operation

The assembled two-chamber MFC is illustrated in supplementary materials (Additional file 1: Fig. S1). It was made of neoprene rubber sheets along with acrylic sheets. In all the experiments, Graphite rods were used as electrodes for the anode and cathode compartments, and both electrodes were connected to titanium wire with a 1 mm diameter (Temco RW0474, USA). The optimized operating conditions were established as described earlier [33]. The anode and cathode chambers were separated by a cation exchange membrane (CEM; NAFION 117), as explained earlier [34], and a synthetic medium was used to make the anolyte. The catholyte contained 100 mM of acidified potassium dichromate to adjust the pH. The distance between the anode and the cathode was approximately 4 cm. Using the same anaerobic sludge from the anaerobic digestion section, 100 mL of anolyte was inoculated in the anode chamber under mesophilic conditions. The electrodes were connected with varied external resistances (R) in the range of 10,000–10 Ω [35]. Experiments were conducted at a temperature of 37 ± 2 °C with shaking at 100 rpm using a Controlled Environment Incubator Shaker (New Brunswick Scientific Co., Edison, N.J., USA). The MFC voltage and current were measured daily using a multimeter (model: UNI-T UT33C +).

#### Evaluation of microbial fuel cell performance

The microbial fuel cell performance was evaluated after the system was stabilized, as evidenced by cyclic voltammetry, as reported previously [33]. Cyclic voltammetry (CV) was carried out using a potentiostat (Autolab Potentiostat Galvano), while 2.1.4 software (Herisau, Switzerland) was used for data acquisition. In CV, the anode graphite rod was the working electrode (7 cm × 1 cm × 0.5 cm), and the cathode rod was the counter electrode, while Ag/AgCl (Hanna instrument, Woonsocket, Rhode Island, United States) was the reference electrode in the anode chamber. The electrochemical behavior was evaluated at different scanning rates (10, 25, 50, and 100 mV/s) based on the recommended range from zero to 100 mV/s [36, 37].

Two types of internal resistance (Rs) were tested in an MFC: a) the charge transfer resistance relates to high frequency, which is represented by the diameter of the semicircle on a Nyquist plot, and b) the diffusive resistance of the electrolyte in the electrode, which hinders the transfer of charges from the solution to the electrode, is represented by the straight line at low frequencies [38]. Electrical impedance spectroscopy (EIS) parameters were adjusted at an AC signal of 10-mV amplitude with a frequency range from 100 kHz to 1 MHz. For MFC studies, 1 or 5 MHz frequencies are sufficient as the lower limit for providing accurate information. Therefore, they complement EIS with other analytical techniques like cyclic voltammetry and biochemical assays [35]. Measurements were conducted at room temperature at atmospheric pressure with a frequency from 0.01 Hz to 100 MHz and a voltage ranging from zero to 1000 mV. The same software was used for data acquisition. The specific capacitance was calculated using the following Eq. (1):

1 $$cp=\frac{I\times \Delta t}{m\times \Delta V}$$

C_sp_ is the capacitance (F/g), *I* (A) is the applied current, *Δt* (s) is the discharge time, *ΔV* (V) is the potential drop, and m (g) is the weight of the graphite electrode. The energy density (Eg) and power density (Pg) were calculated from galvanostatic charge–discharge GCD measurements through $\mathrm{energy\,density }({{\text{E}}}_{{\text{g}}})= \frac{1}{2}*{\text{Csp}}*(\Delta {\text{v}}{)}^{2}$where Eg is the energy density (Wh/kg).$power\,density ({P}_{g)}= \frac{{E}_{g}}{\Delta t}$where Pg is the power density (W/kg), and Δt (s) is the discharge time in seconds.

Once the MFC was stable, chicken feather hydrolysate was tested as a carbon carrier in the anolyte. This was done using acidified dichromate as a catholyte at a pH of 2. The biologically treated feather hydrolysate used in this study was obtained after solid-state fermentation of chicken feathers using a recombinant *Bacillus subtilis* strain. In this experiment, the voltage and current were monitored daily, while COD was monitored using HANNA COD reagent set HI93754B-25 (Wonnsocke, USA) based on the dichromate method, which was adapted from the standard EPA 410.4 and ISO methods for determination of COD with expected concentrations ranging from 0 to 15,000 mg/L O_2_ [39].

### Microbial electrolysis cell

#### Microbial electrolysis cell construction

**Fig. 1:**
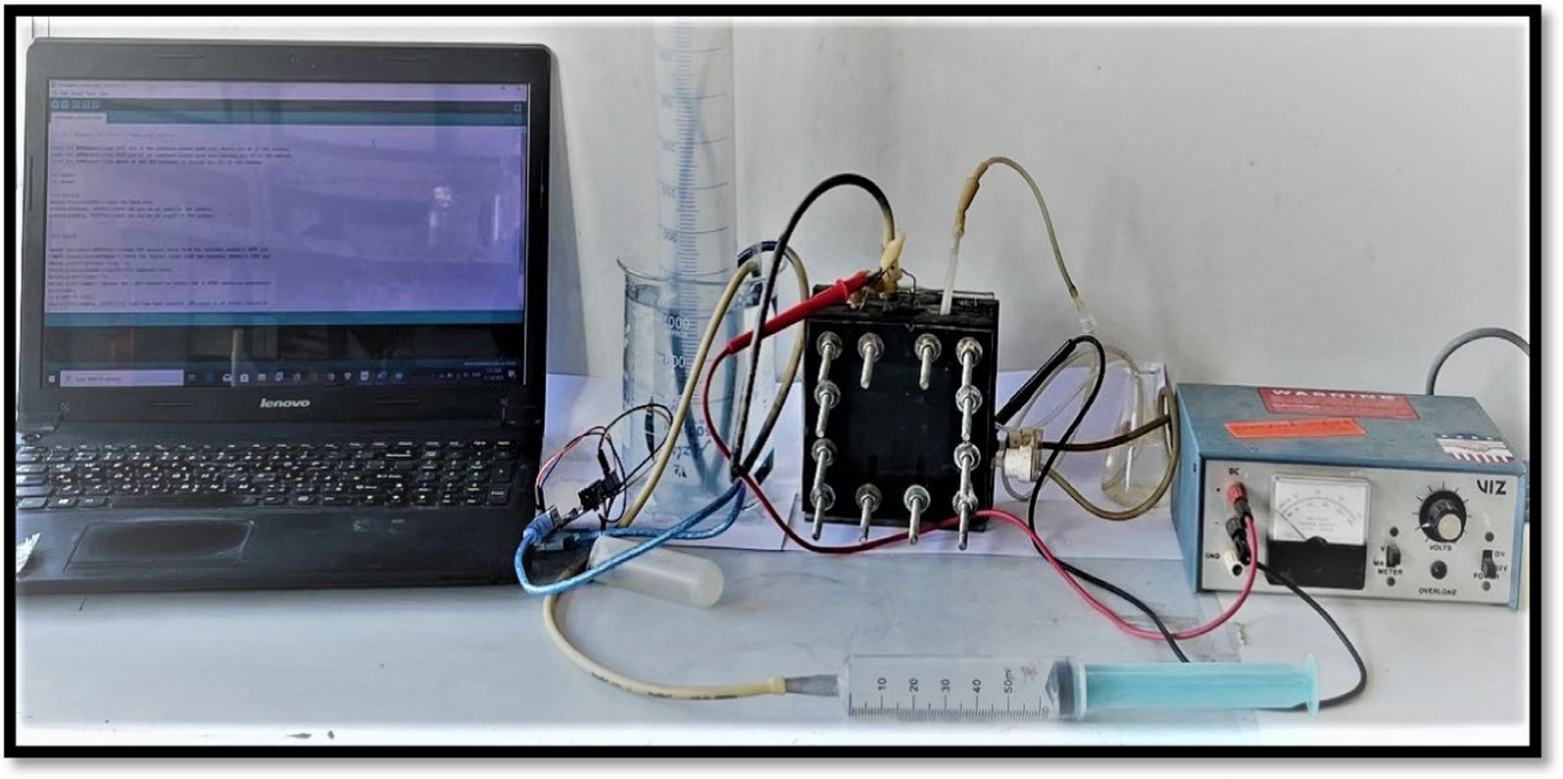
Microbial electrolysis cell constructed with a hydrogen gas collection system

The dual-chamber compact cubic microbial electrolysis cell used in this study was similar to the MFC, with some modifications in the cathode compartment (Fig. 1). A graphite rod (7 cm × 1 cm) was used as the anode. Carbon cloth supported on stainless steel mesh (5 cm × 5 cm) that is coated with platinum (Pt) (0.5 mg Pt/cm^2^) was used as the cathode, where it was the catalyst for the oxygen reduction reaction in the cathode chamber. Both the anode and cathode were connected externally using titanium wire in the presence of an in-between external resistance (varying from 10 kΩ to 10 Ω). The anode and cathode chambers were separated by the same type of cation exchange membrane used in MFCs. For continuous hydrogen gas collection, the water displacement method was used, and the yield was detected using a hydrogen sensor (MQ8) [18, 40] (http://www.learningaboutelectronics.com/Articles/MQ-8-hydrogen-sensor-circuit-with-arduino.php).

#### Microbial electrolysis cell operation

The microbial electrolysis cell was operated at room temperature of ~ 25 °C under static conditions and in batch mode, with each cycle lasting 24–48 h. The anode chamber of the cell was filled with the same anaerobic sludge provided in the MFC mentioned earlier and fed with a synthetic medium as described earlier [41]. The catholyte was prepared with a 50-mM phosphate buffer solution consisting of K_2_HPO_4_ and KH_2_PO_4,_ pH = 7.6. The MEC started with external power using a power supply at a voltage of 0.8 V. Current and voltage were monitored daily using a digital voltammeter. Once the acetate-fed MEC was stabilized, feather hydrolysate was introduced for its ability to run the MEC for hydrogen production; acetate was replaced by hydrolysate, resulting from keratinous waste biodegradation.

#### Monitoring of hydrogen gas production

The water displacement method was used for hydrogen gas collection, and the volume was measured using a graduated cylinder. The hydrogen yield was estimated based on previous studies [18, 42].

$$\mathrm{Yield\,of\,hydrogen\,gas\,produced}=\frac{\mathrm{numbe\,of\,moles\,of\,hydrogen\,gas\,produced}}{\mathrm{Number\,of\,moles\,of\,substrate\,consumed}}$$

Since P. V = n.R.T., accordingly,$$\mathrm{the\,number\,of\,moles\,of\,hydrogen\,gas\,produced}= \frac{{\text{P}}.{\text{V}}}{{\text{R}}.{\text{T}}}$$

- V is the volume of hydrogen produced (mL).
- P is the atmospheric pressure (bar).
- R is the universal gas constant (8.314 *10^–2^ L.bar/K.mol).
- T is the absolute temperature (K).
  $$\mathrm{No\,of\, moles\,of\,substrate\,consumed}=\mathrm{ Change\,in\,COD\,of\,substrate }(\Delta {\text{COD}})$$

Accordingly,$$\mathrm{Yield\,of\,hydrogen\,gas\,produced}=\frac{{\text{P}}.{\text{V}}}{{\text{R}}.{\text{T}}. \Delta {\text{COD}}}$$

In MEC, the theoretical molar yield of hydrogen utilizing acetate as a substrate is 4 mol/mol acetate. In addition, a practical molar yield as high as 3.7 mol/mol (93%) was reached when the applied voltage was 0.8 V, as reported earlier [42, 43].

## Results

### Elemental composition analysis of feather hydrolysate

A good approach to making chicken feathers easier to digest is to treat them biologically with microorganisms first. As a result, their complex structure has degraded, making them useful as a raw material for biogas production. Chicken feather hydrolysate was obtained using either SSF with or without wheat bran. Feathers have a relatively low C/N ratio. Therefore, to ensure an optimal carbon-to-nitrogen ratio when pretreating feathers to test their availability for biogas production anaerobically, the elemental composition of feather hydrolysate was investigated using SEM–EDX. The major elements found in all the samples were C, N, O, P, S, Cl, and K. The data revealed that the net C/N ratio for the hydrolysate of SSF was twice as high as that for SSF (WB) (Additional file 1: Table S1).

### Anaerobic digestion experiments for methane production

The data showed that the AD treatment, in which FH was fed as an organic matter source, produced the highest methane yield (0.67 Nm^3^/kg substrate), which was 2 and 3 folds greater than that obtained when using either acetate or untreated feather particles, respectively (Table 1). Chicken feather hydrolysate significantly improved the efficiency of the all-over anaerobic process, with a COD removal percentage reaching 86% (Table 2).

**Table 1:** Production of methane and hydrogen gases from anaerobic digestion

Treatment using different substrates	Gas yields from anaerobic digestion	
	Methane (Nm^3^/ kg dry substrates)	Hydrogen (mmol/day.L)
Acetate (Control)	0.40 $\pm$ 0.01	0.42 $\pm$ 0.015
Untreated chicken feather	0.20 $\pm$ 0.02	0.07 $\pm$ 0.02
Feather hydrolysate	0.67 $\pm$ 0.01	0.85 $\pm$ 0.01

Experiments were carried out in triplicate, and the results are expressed as the mean $\pm$ standard deviation

**Table 2:** COD monitoring and COD removal efficiency in the MFC powered with feather hydrolysate after 27 days

Day	COD (mg/l)	COD removal (%)
1	5828 $\pm$ 51.58	8.00
7	4682 $\pm$ 53.30	20.00
10	2353 $\pm$ 51.51	60.00
12	2105 $\pm$ 27.53	64.00
14	1881 $\pm$ 53.10	68.00
17	1500 $\pm$ 76.37	74.30
21	1180 $\pm$ 52.91	80.00
24	1032 $\pm$ 41.32	82.00
26	965 $\pm$ 5.00	83.40
27	838 $\pm$ 26.10	86.00

Experiments were carried out in triplicates, and the results are expressed as the means $\pm$ standard deviation

### Anaerobic digestion for hydrogen production

The potential of using biologically treated FH as a feedstock for hydrogen production in anaerobic digestion was also tested. Adding a 2-bromoethanesulfonate inhibitor to the anaerobic digestion reactors directed the reaction toward hydrogen production, but the production rates varied depending on the substrates used. The highest hydrogen gas yield was 0.85 mmol/day.L was acquired from treated chicken feathers (FHs). A lower gas yield was recorded when acetate was used as a substrate, corresponding to 0.42 mmol/day.L, and the lowest yield was evident in the presence of untreated FP as the substrate (Table 1).

### Evaluation of microbial fuel cell performance

The cells were compact systems made of neoprene rubber sheets, with a cation exchange membrane separating both chambers. Voltage and current were monitored daily on all trials using a digital multimeter for 55 days (Additional file 1: Fig. S2). Results showed that electricity generation was most significant when the lowest resistance (10 Ω) was applied as the voltage reached 811 mV and 1.1 mA after 42 days of running the experiment.

**Fig. 2:**
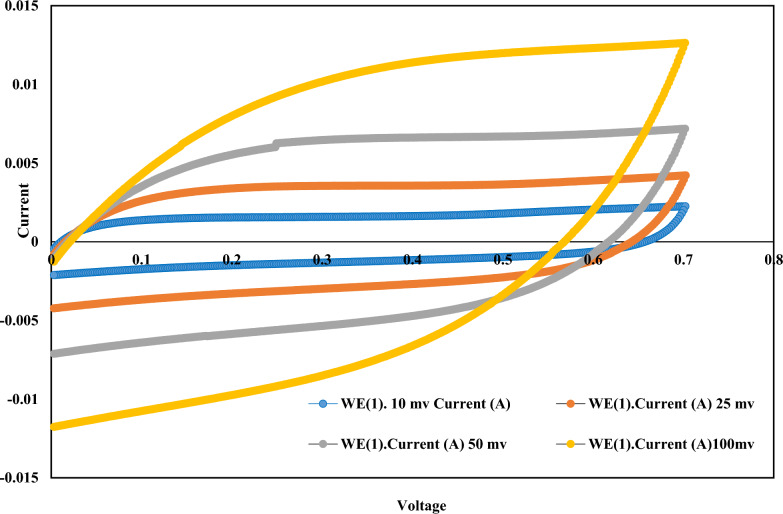
Cyclic voltammograms obtained at different scan rates using a graphite rod anode

**Fig. 3:**
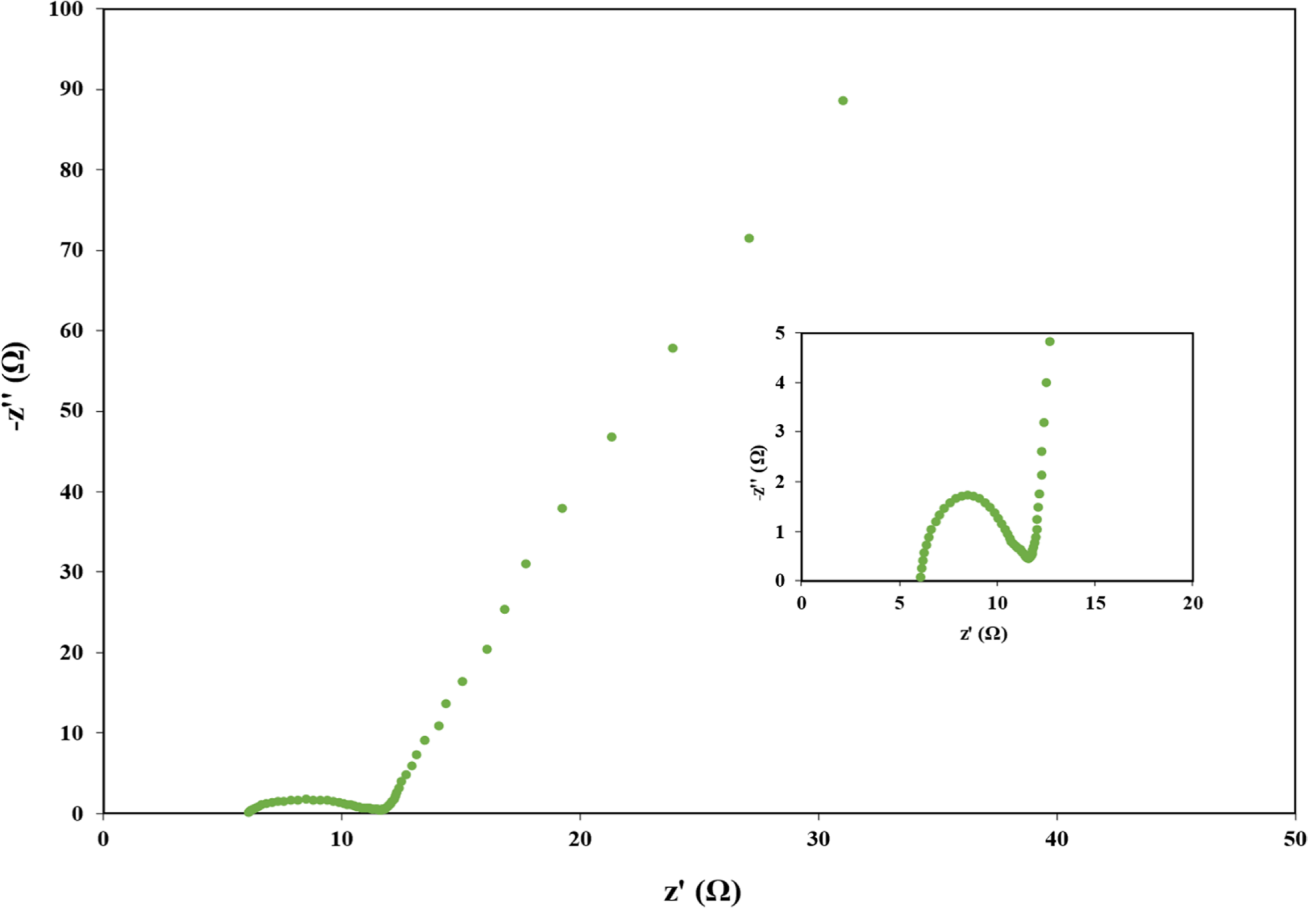
Electrochemical impedance spectroscopy of MFC-acetate

Cyclic voltammetry was performed at different scan rates of 10, 25, 50, and 100 mV/s, as shown in Fig. 2. Electrical impedance spectroscopy (EIS) was used to determine the electrochemical properties of the MFC. Figure 3 shows the electrical impedance of the MFCs recorded in the frequency range of 0.01 Hz–100 kHz. Cyclic voltammetry (CV) patterns were used to determine the relationship between the current and voltage at different scan rates. At a lower scanning rate (10 mV/s), an oxidation peak was observed between 0.1 and 0.2 V, with a reduction peak between 0.4 and 0.5 V. Higher scanning rates of 25, 50, and 100 mV/s yielded similar patterns, indicating the electrodes' stability at higher rates than 10 mV/s.

**Fig. 4:**
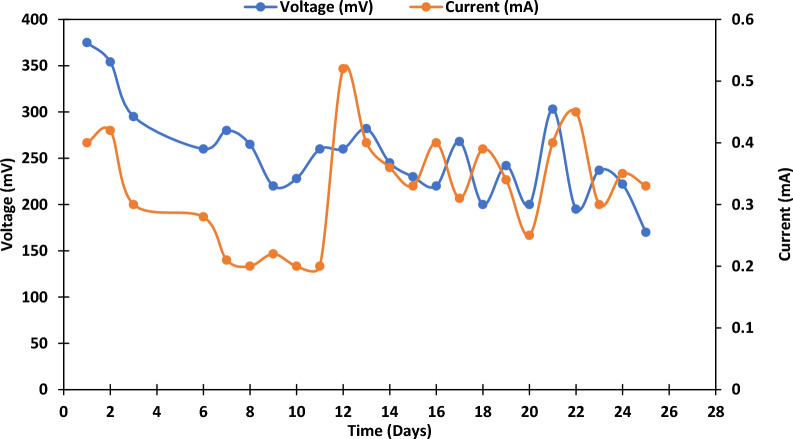
Monitoring voltage and current for feather hydrolysate-fed MFC

The specific capacitance Csp was calculated from galvanostatic charge–discharge (GCD) curves according to the equations mentioned in the Materials and Methods. It was 121.79 F/g when the applied current was 2 amperes, the discharge time was 42 s, and the potential drop was 0.69 V, with a negligible value of electrode mass. Consequently, the power and energy density were 0.69 W/kg and 29.28 Wh/kg, respectively. After feather hydrolysate addition, the highest voltage was 400 mV, and the highest current was 0.7 mA (Fig. 4). Table 2 shows the COD monitoring and removal efficiency. The obtained data showed the cell's high ability to run on chicken feather hydrolysate to produce electricity with COD removal efficiency, reaching 86% after 27 days.

### Microbial electrolysis cell performance for biohydrogen production

**Fig. 5:**
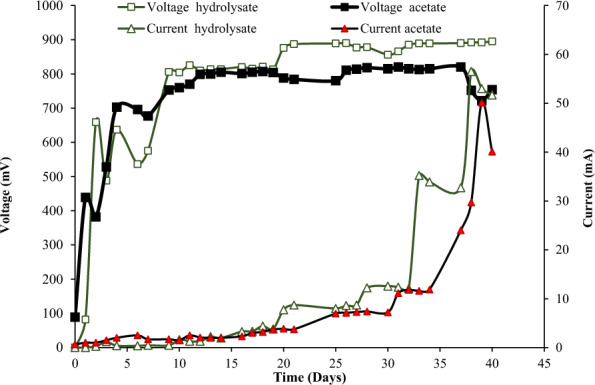
Voltage and current monitoring of control acetate-MEC versus feather hydrolysate-MEC

Figure 5 indicates an increase in the cell potential at first, followed by a stabilization of the potential between 750 and 820 mV. Initially, a higher external resistance resulted in minimal electron demand when an external resistance (ranging from 10 kΩ to 10 Ω) was placed between both electrodes. A similar was reported earlier [21]. The calculated power density (power per cathode area), based on the maximum current density (electrical current per cathode area) and its corresponding voltage, was 7.23 W/m^2^ (Additional file 1: Fig. S3). During the electrohydrogenesis process, the current production was directly proportional to the hydrogen gas production under anaerobic conditions [44]. The MQ-8 sensor detected hydrogen gas production and its purity after being calibrated using hydrogen gas (99.999% purity). The produced hydrogen was collected through the water displacement method with a maximum hydrogen production volume of 400 mL during MEC incubation of 42 days. The acquired production rate was 4.4 mmol/day.L. The equation mentioned in the experimental section was used to calculate the hydrogen production yield, resulting in 3.55 mol/mol of substrate, which corresponds to 88.97% of the theoretical production yield (4 mol/mol) and 95.95% of the highest practical yield of 3.7 mol/mol when a voltage of 0.8 V was applied for COD removal, equivalent to 96%. Higher current density led to an expected increase in production yield, showing a synergistic correlation between the two factors.

**Fig. 6:**
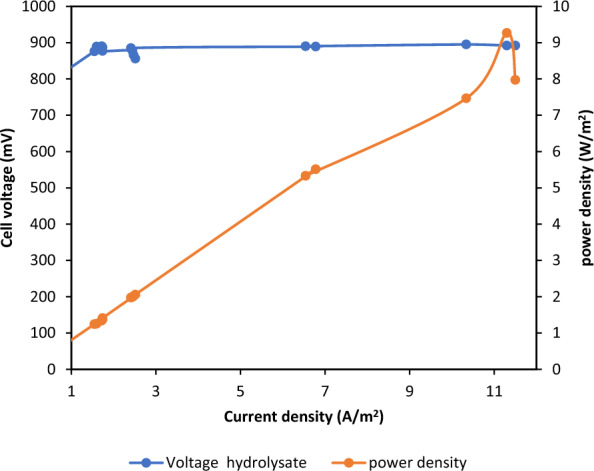
Determination of the current and power density of MECs powered by feather hydrolysate

It can be concluded that the constructed cell is an efficient microbial electrolysis cell (MEC) that can produce a high yield of hydrogen with a higher current density. Figure 5 shows that the highest voltage and current measured in the MEC test using the feather hydrolysate were 895 mV and 56.5 mA (A/m^3^), respectively. These values are slightly higher than those obtained from the acetate MEC with the same applied voltage (0.8 V). Furthermore, the hydrogen yield was monitored at the end of the experiment using the mq-8 sensor, as previously mentioned. MEC powered by feather hydrolysate produces hydrogen at a rate of 7.5 mmol/day.L and 92.66% COD removal efficiency, with a power density of 9.266 W/m^2^ and a current density of 11.5 A/m^2^ (Fig. 6).

## Discussion

EDX analysis results agree with previous reports analyzing chicken feather waste indicated that feather hydrolysate from SSF, supplemented with wheat bran, improved its applicability for microbial energy production compared to the hydrolysate alone. These results are consistent with previous recommendations concerning the co-digestion of pretreated feather waste with other co-substituents to enhance its potency for anaerobic fermentation. A previous study recommended optimizing the first biodegradation step of keratinous waste to produce the ideal fermentation broth for the next biofuel production step [45, 46].

Results of the anaerobic digestion showed higher methane yields because of the recombinant strain and how it improved the feather hydrolysate properties. Generally, the biogas yield from different substrates depends on several factors, such as the microbial community composition, process parameters, and mixing feedstocks [47]. Consequently, the presence of specific keratin-degrading bacteria can maximize biogas production through efficient digestion of proteins and lipids in keratin biomass, which could yield more methane per kg of substrate than acetate [48]. The present findings demonstrate the recombinant bacterial cells' ability to use feather waste as a sole source of nutrients. Rich organic matter is recommended to induce methane gas production. It has been reported that this strain of *B. subtilis* has a multicopy recombinant plasmid (p5.2) that has 20 times more of the complete alkaline protease gene than the native wild type of the microorganism [27]. According to previous studies, *B. subtilis* DB100 (p5.2) displays superior feather degradation ability, with the highest levels of proteolytic and keratinolytic activity [6].

The biologically treated FH was reported to be rich in free amino acids (approximately 37.5 µmole leucine/mL) and has a high level of soluble proteins (214 mg/g substrate) [27]. This microbe is a facultative aerobe and exists in the second step, maximizing methane production. This strain played a fundamental role in the feather hydrolysis stage and would continue providing essential methane production nutrients. Using anaerobic digestion with FH is a low-cost alternative, as well as waste valorization. These findings align with earlier research on the key determinants affecting keratin-rich waste generated by the keratinase-producing *Bacillus subtilis* recombinant strain [26]. Another study showed that feathers treated with recombinant *Bacillus megaterium* had twofold greater biogas production than wild *B. megaterium* [30].

Previous reports confirm that fermentation broth produced from keratin waste material via bacterial degradation is a suitable feedstock producing more hydrogen than the standard Bacto-Peptone [49]. The prior step of keratin biodegradation facilitates the subsequent anaerobic fermentation compared to untreated chicken keratin waste, a recalcitrant, insoluble protein. The FH treatment showed an enhanced hydrogen production rate compared to the control due to the increased protein concentration.

The tested electrochemical properties of the microbial fuel cell performance confirmed that the electrode used had higher electrogenic activity and a faster reaction rate in the anode chamber, which aligns with what other research has found [33, 38]. The voltammograms indicated faster scanning rates and a higher potential window of 100 mV/s, resulting in higher currents than lower potentials. Previous reports [36] confirm faster scan rates increase electron transfer, leading to higher currents

A peak was observed, indicating the reduction of dichromate at the cathode at the scanning rate of 10 mV/s. At lower scanning rates (10 mV/s and 25 mV/s), a reduction peak was observed, indicating a complete reduction in dichromate with a potential of 0.6–0.7 V, during which a steady state was reached; this effect, known as the faradic effect, was eliminated at higher scanning rates.

As reported earlier [33], there are many differences in the cyclic voltammograms of MFCs, which may depend on the use of different electrode materials, the application of different scanning features, the conductivity of the organic substrate, and the anolyte. At low frequencies, the MFC exhibited a high diffusive resistance, indicating the presence of slow bioelectrochemical substrate oxidation-reduction processes. A different study is discussed differently: A cyclic voltammogram confirms the clear peaks for oxidation and reduction reactions that were seen from the peak current because modified carbon xerogel was involved in redox reactions. As the scan rate increased, the CV showed an increase in redox peak currents, as expected for reversible reactions. The cyclic voltammetry results suggest that the modified carbon xerogel is active in redox reactions in the cathode chamber [50].

The CV data and Nyquist diameter (Fig. 3) indicate a charge transfer resistance of approximately 6 ohms. This resistance was measured under different external resistances in the range of 10 to 10,000 Ω. Lower Rs values improved the overall conductivity of the MFC cell, reflecting a rapid rate of electron transfer between the anode and the cathode.

This study reports that chicken feather hydrolysate could be used as a carbon carrier in a microbial fuel cell for electricity generation. These data are in agreement with a previous study in which chicken feather waste was used to produce electricity with a maximum voltage of 141 mV using *Pseudomonas aeruginosa*, reached a maximum power density of 1206.78 mW/m^2^ and a maximum current density of 8.6 mA/m^2^ after 14 days of incubation [51]. Moreover, the maximum power density of 379 ± 8 mW/m^2^ was produced from sesame seed cake media with inoculum of mixed consortia from lake sediment. An electrogenic bacterial species, *Kluyvera georgiana* MCC 3673, was previously isolated by enrichment in microbial fuel cells (MFC) using oilseed cake as a growth substrate [16].

In MEC-acquired data (Fig. 5), the electrical current was initially low and increased to a maximum value of 51 mA when the resistance decreased to 10 Ω after 36 days of incubation. These data are in agreement with previous reports concerning an increase in electrical current with a decrease in external resistance [18, 21]. The obtained current (51 mA, 450 A/m^3^) represents 1.54 times the reported maximum current density (292 A/m^3^) using the same applied voltage (0.8 V) [42]. The higher results, when compared to those of other reactors at the same applied voltage (0.8 V) and substrate concentration (1 g/L acetate), could be attributed to differences in reactor design, microbial culture, and incubation conditions [52]. In addition to current and voltage monitoring, the current and power densities were calculated to describe cell performance. The data show the relationships between the cell potential and current density, as well as between the cell potential and power density (Fig. 6). The results also show that the highest current density was 10.2 A/m^2^, similar to what other research has found when using a synthetic medium with acetate as a substrate in MEC to produce hydrogen [43, 53, 54]. Consequently, feather hydrolysate can successfully serve as an effective substrate for hydrogen production in MECs with lower energy consumption than acetate (2.17 J). This is the first report to demonstrate the use of chicken feather hydrolysate as a substrate in MECs.

## Conclusions

A two-stage fermentation system has shown that the treated feather hydrolysate from *B. subtilis* is promising for bioenergy production. Feather hydrolysate yields more methane and hydrogen gas in anaerobic digestion than acetate or untreated feathers. In MFCs, feather hydrolysate supplemented with wheat bran showed improved performance with high power density and low diffusive resistance. This is the first report of the successful utilization of chicken feathers along with wheat bran as a substrate for hydrogen production. Microbial electrolysis cells yielded nine times more hydrogen than anaerobically digested feather hydrolysate. This makes MECs a promising low-cost technology for producing biohydrogen from keratin-rich wastes.

### Supplementary Information

**Additional file 1: Figure S1.** Constructed dual chamber microbial fuel cell. **Figure S2.** Voltage and current monitoring for acetate fed MFC. **Figure S3.** Current density and power density monitoring versus voltage results in acetate MEC. **Table S1.** Comparison between chemical composition between feather hydrolysate resulted using submerged fermentation or solid-state fermentation.

## Data Availability

All data generated during this study are included in this article and the additional files. Raw data are available on reasonable request.

## Abbreviations

Csp: Specific capacitance
CV: Cyclic voltammetry
EDX: Energy dispersive X-ray analysis
Eg: Energy density
FH: Feather hydrolysate
FP: Untreated Feather intact particles
MEC: Microbial Electrolysis Cell
MFC: Microbial Fuel Cell
MFC-acetate: Microbial fuel cell powered by acetate
MFC-FH: Microbial fuel cell powered by prior biologically treated feather hydrolysate
Pg: Power density
WB: Wheat bran
