# Supplementary material for: Bioenergy production from chicken feather waste by anaerobic digestion and bioelectrochemical systems
Source: Microb Cell Fact. 2024 Apr 4;23:102. doi: 10.1186/s12934-024-02374-5 (PMC10996200; doi:10.1186/s12934-024-02374-5)
Supplement: Supplementary file 1 — Additional file 1: Figure S1. Constructed dual chamber microbial fuel cell. Figure S2. Voltage and current monitoring for acetate fed MFC. Figure S3. Current density and power density monitoring versus voltage results in acetate MEC. Table S1. Comparison between chemical composition between feather hydrolysate resulted using submerged fermentation or solid-state fermentation. [file 12934_2024_2374_MOESM1_ESM.docx]

**Additional file 1**

**Figure S1. Constructed dual chamber microbial fuel cell**

**Figure S2. Voltage and current monitoring for acetate fed MFC**

**Figure S3. Current density and power density monitoring versus voltage results in acetate MEC**

**Table S1: Comparison between chemical composition between feather hydrolysate resulted using submerged fermentation or solid-state fermentation**

| **Element** | **Feather hydrolysate results from SSF ^1^**  **(Mass%)** | **Feather hydrolysate results from SSF ^2^ B (Mass%)** |
| --- | --- | --- |
| **C** | 36.87 ±0.30 | 38.72±0.26 |
| **N** | 13.03 ±0.47 | 8.28±0.34 |
| **O** | 35.75±0.53 | 47.14±0.55 |
| **P** | 0.38 ±0.05 | 0.54±0.04 |
| **S** | 2.45 ±0.07 | 0.36±0.03 |
| **Cl** | 7.13 ±0.10 | 3.10±0.08 |
| **K** | 0.98 ±0.06 | 1.87±0.07 |
| **Total** | 100.00 | 100.00 |

1. Solid state fermentation of feather hydrolysate (SSF)

2. Solid-state fermentation of feather hydrolysate supplemented with wheat bran (SSF (WB))
